# Detection of HPV RNA molecules in stratified mucin-producing intraepithelial lesion (SMILE) with concurrent cervical intraepithelial lesion: a case report

**DOI:** 10.1186/s12985-019-1180-2

**Published:** 2019-06-03

**Authors:** Shiho Fukui, Kazunori Nagasaka, Naoko Iimura, Ranka Kanda, Takayuki Ichinose, Takeru Sugihara, Haruko Hiraike, Shunsuke Nakagawa, Yuko Sasajima, Takuya Ayabe

**Affiliations:** 1 Department of Obstetrics and Gynecology, Teikyo University School of Medicine, Tokyo, Japan; 2 Gynecology Center, Sanno Hospital, International University of Health and Welfare, Tokyo, Japan; 3 Department of Pathology, Teikyo University School of Medicine, Tokyo, Japan

**Keywords:** Stratified mucin-producing intraepithelial lesion, Cervical intraepithelial lesion, Cancer stem cell, Human papillomavirus

## Abstract

**Background:**

Stratified mucin-producing intraepithelial lesion (SMILE) is a rare precursor lesion in the uterine cervix that is considered a variant of adenocarcinoma in situ (AIS). Although human papillomavirus (HPV) is thought to be related to the development of SMILE, there is little information available on the detection of HPV integrated into the lesion.

**Case presentation:**

A 30-year-old female underwent a routine uterine cervical cancer screening, and her Pap smear indicated the possible existence of atypical glandular cells. A cervical biopsy with endocervical curettage was performed. The histopathological analysis showed that she had SMILE and high-grade squamous intraepithelial lesion (HSIL) on her cervix. The lesion was found to be positive for HPV genotypes 52 and 68 by multiplex PCR. In situ hybridization with HPV RNA probes revealed that these HPV types were involved in the onset of HSIL and SMILE, respectively.

**Conclusions:**

Rare, high-risk HPV genotypes may contribute to the development of SMILE, and their detection can be useful for preventing the progression to carcinoma and ensuring adequate patient management.

## Background

Uterine cervical cancer is the second most commonly diagnosed cancer and the third leading cause of cancer death among women in developed countries [1]. Although cervical screening including the human papillomavirus (HPV) test has reduced the incidence and mortality rate of cervical cancer worldwide [2], there is still little information about the role of less prevalent and rare HPV genotypes, such as HPV68, during cervical carcinogenesis [3].

Stratified mucin-producing intraepithelial lesion (SMILE) is an uncommon premalignant lesion of the uterine cervix [4]. It is thought to arise from the reserve cells of the transformation zone throughout the full epithelial thickness of a lesion, with some overlap with the architecture of squamous intraepithelial lesion (SIL) or adenocarcinoma in situ (AIS) [4]. SMILE is characterized by several histopathological features, including epithelial stratification, diffuse mucin production throughout the epithelial layers, and an absence of classic gland formation [5]; nuclear atypia, hyperchromasia, mitosis, and apoptotic bodies are often observed in the lesion, which is similar to other forms of intraepithelial neoplasia including usual-type AIS of the endocervical glandular epithelium. Histochemical staining for mucin [6–8] and immunohistochemical detection of Ki-67/Mindbomb E3 ubiquitin protein ligase (MIB)-1 have revealed a high proliferative index [4]. Importantly, diffuse positivity for the cell cycle regulation protein p16INK4a—which is associated with high-risk HPV infection—is also observed [9]; however, there is limited information available on the involvement of high-risk HPV in the pathogenesis of SMILE [10–12].

Studies over the last two decades have shown that persistent HPV infection is the main cause of cervical cancer development. Clinically validated HPV tests are recommended by the U.S. Preventive Services Task Force (USPSTF) and the Japan Society of Obstetrics and Gynecology for cervical pre-cancer screening, triage, and treatment follow-up in clinical practice [13, 14]. About 40 different HPV types can infect the cervix, of which 14 (type 16, 18, 31, 33, 35, 39, 45, 51, 52, 56, 58, 59, 66, and 68) are classified by the World Health Organization as being associated with a high risk of SIL and cervical cancer development [15–17]. Most oncogenic or high-risk HPV types associated with invasive cervical cancer are phylogenetically clustered within the species groups *Alphapapillomavirus* 9 (Alpha-9: HPV16 along with HPV31, 33, 35, 52, and 58) or *Alphapapillomavirus* 7 (Alpha-7: HPV18 along with HPV39, 45, 59, and 68) [18]. These two groups account for approximately 75 and 15%, respectively, of all cervical cancer cases worldwide [19]. However, compared with HPV16 and HPV18, the carcinogenicity of other HPV types has not been well investigated, and rare HPV genotypes are poorly understood. It is thought that high-risk HPVs preferentially infect and replicate in the basal layer of the epithelium [20] with the integration of HPV sequences into the host cell genome leading to SIL progression. On the other hand, it is unclear whether high-risk HPV contributes to the development of SMILE [21]. HPV RNA in situ hybridization (ISH) is an established method for detecting genomically integrated HPV sequences [22, 23]. In the present work, we investigated whether rare, high-risk HPV contributes to the development of SMILE using RNA ISH to assess the integration of viral DNA in cervical cancer lesions.

## Case presentation

### Case report

**Fig. 1:**
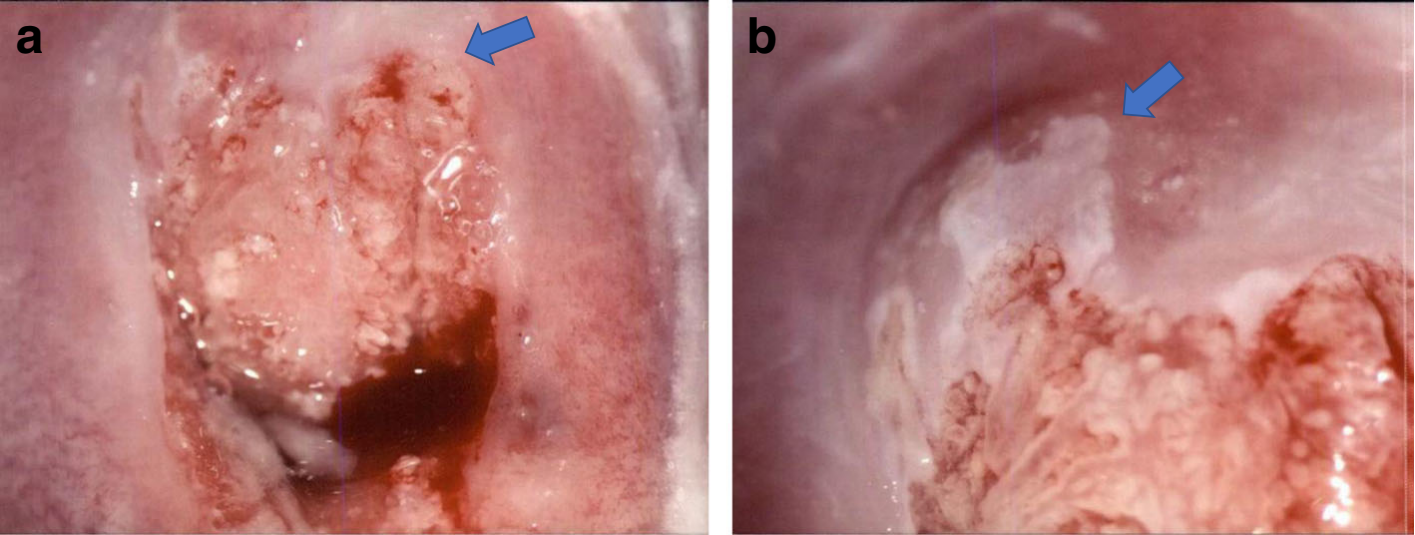
Colposcopic examination of the cervical lesion in the patient**. a.** Smooth, white, dense lesions at the 1 o’clock direction (arrow) and **b.** a coarse mosaic at the 11 o’clock direction (arrow) can be seen

**Fig. 2:**
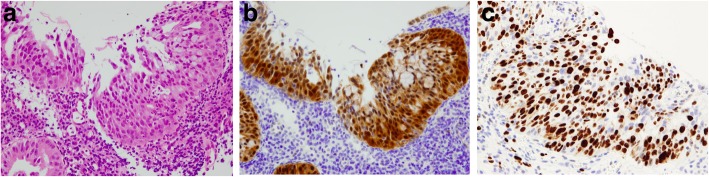
Histopathological examination. **a** Histopathological examination of SMILE. The lesion comprised heterotypic cells staining positive for mucin. **b**, **c** Immunohistochemical detection of p16INK4a (b) and MIB-1 (Ki-67) (c) in a SMILE revealed diffusely positive and positive staining, respectively, throughout the epithelial layer

A 30-year-old female (gravity 0, parity 0) was referred to our hospital for routine uterine cervical cancer screening, and her Pap smear indicated the possible existence of atypical glandular cells. A colposcopic examination revealed dense white lesions in the 1 and 11 o’clock directions (Fig. 1a, b). Punch biopsies were performed after the colposcopic examination. Histopathological analysis of punch biopsies showed a SMILE on the cervix (Fig. 2a) as well as extensive immunopositivity for Ki-67, which is consistent with previous reports that cells undergoing endocervical differentiation are neoplastic and not entrapped benign columnar cells [4] (Fig. 2b). The involvement of HPV in the development of SMILE was also suggested by the positive p16 staining (Fig. 2c). The lesion was found to be negative for HPV genotypes 16, 18, 45, 31, 33, 35, 39, 45, 51, 56, 58, 59, and 67 but positive for HPV genotypes 52 and 68 by multiplex PCR.

**Fig. 3:**
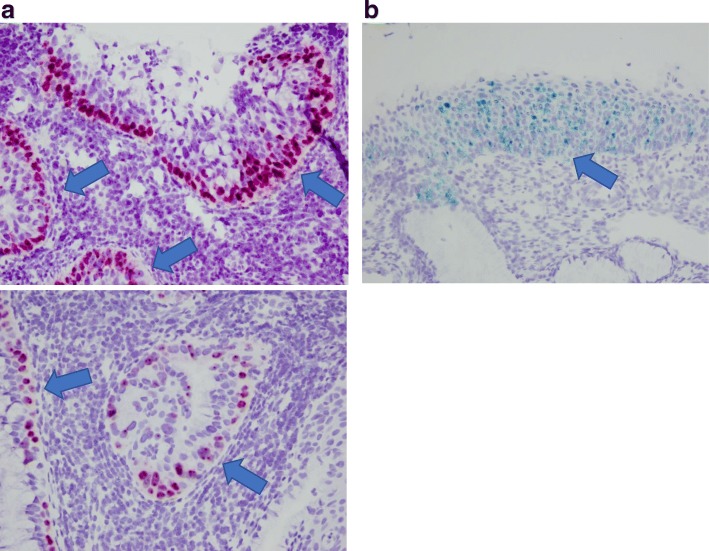
RNA fluorescent in situ hybridization. Biopsied specimens obtained from the 11 o’clock direction of the cervix. These specimens were diagnosed as SMILE and high-grade squamous intraepithelial lesion (HSIL). **a** Basal and parabasal SMILE cells were positive by ISH analysis using a probe for HPV type 68 (red: arrows). **b** All layers in the epithelium of HSIL showed a positive signal by ISH using a probe for HPV type 52 (blue: arrow)

We next examined lesions by single-molecule RNA fluorescent ISH using the RNAscope system (Advanced Cell Diagnostics, Newark, CA, USA) [22] and specific RNA probes targeting HPV 52 (catalog no. 311611) and 68 (catalog no. 478631-C2). Frozen cervical tissue sections (10 μm thick) were fixed with 4% paraformaldehyde in phosphate-buffered saline for 15 min at 4 °C, dehydrated by serial immersion in 50, 70, and 100% ethanol for 5 min each at room temperature, and treated with protease for 30 min at room temperature. The probes were then hybridized for 2 h at 40 °C, followed by RNAscope amplification. The sections were labeled with conjugated wheat germ agglutinin (Thermo Fisher Scientific, Waltham, MA, USA) diluted 1:100 to detect cell borders, and were counterstained with 4′,6-diamidino-2-phenylindole, according to the manufacturer’s instructions. Images were acquired on an LSM 510 META confocal microscope (Zeiss, Oberkochen, Germany). HPV 68 RNA was detected in the lower epithelial layers of the SMILE along with cytoplasmic mucin (red: Fig. 3a). Notably, all basal lesions in the SMILE were positive for HPV type 68 based on ISH analysis using the corresponding probe (Fig. 3a, upper and lower images). The stratified epithelium had architecture similar to that of a high-grade squamous intraepithelial lesion and was positive for HPV 52 (blue: Fig. 3b). The patient underwent conization of the uterine cervix, and since the surgery there has been no evidence of abnormal cytology.

## Discussion and conclusions

Histologically, SMILE is characterized by a multilayered atypical epithelium composed of cells with intracytoplasmic mucin in all cell layers. SMILEs are p16 positive and have a high MIB-1 proliferation index. However, SIL and AIS may coexist with SMILE, which is not surprising given their association with HPV infection. Moreover, a minority of invasive cervical carcinomas also exhibit both squamous and glandular features (referred to as adenosquamous carcinoma). Apart from the original description, there is little information on SMILEs, especially in relation to high-risk HPV infection [11]. Most SMILEs are classified as atypical glandular lesions since they do not meet all of the criteria of AIS and are characterized by mucin production; it may also be confused with reactive endocervical glandular cells that tend to have finely dispersed nuclear chromatin with prominent nucleoli, in contrast to the cells in SMILE that exhibit increased nuclear density with inconspicuous nucleoli. Notably, our findings provide evidence that SMILE likely arises from rare, high-risk HPV-infected stem or reserve cells with multilineage differentiation potential.

Previous reports have suggested the preferential physical interaction of the virus with squamocolumnar junctions in the transformation zone of the basal epithelium that forms reserve cells with stem cell (SC) properties [24, 25]. These observations, along with technological advances in the identification of cancer (C)SCs based on marker expression, have facilitated the identification and characterization of cervical CSCs. To date, there is no evidence that HPV infection contributes to the development of SMILE due to the scarcity of clinical specimens. Findings from a limited number of cases suggest that the pathologic features of SMILE are related to cervical CSC [10]. If mildly atypical glandular cells are observed in conjunction with a positive HPV test and persist in repeated Pap tests, or are detected in one-time HPV testing, it may be difficult to confirm HPV infection in the basal epithelium since there may be other concurrent cervical abnormalities [12].

Examination of additional cases would be helpful in confirming the existence of CSCs in SMILEs. Therefore, we consider that most high-risk HPV types in cervical cancer are easily detectable, given their diffuse presence in the epithelium compared to that of high-risk HPV types, such as Alpha-7, that are predominant in the basal layer. Furthermore, the case implies that different HPV-infected cells individually define their disease phenotype as HSIL or SMILE. We speculate that SIL, AIS, and SMILE differ in terms of cellular origin with different HPV life cycles. Alpha-7 HPV types such as HPV18 and HPV68 may preferentially remain in the basal epithelium, unlike HPV16-related Alpha-9 HPV types, such as HPV52. In addition, complex cases of multiple HPV infection may exhibit distinct histopathology. Notably, our observations had certain limitations because of the study being a case report. Hence, further studies are needed to explore these possibilities. Nonetheless, our findings provide a basis for investigating multiple infection by Alpha-7 and -9 HPV types, the carcinogenicity of the rare HPV genotypes, and the outcome of SMILE in the cervix.

## Data Availability

Not applicable.

## Abbreviations

AIS: Adenocarcinoma in situ
CSC: Cancer stem cell
HPV: Human papillomavirus
HSIL: High-grade squamous intraepithelial lesion
ISH: In situ hybridization
MIB: Mindbomb E3 ubiquitin protein ligase
SC: Stem cell
SIL: Squamous intraepithelial lesion
SMILE: Stratified mucin-producing intraepithelial lesion
